# Can an artificial intelligence chatbot be the author of a scholarly article?

**DOI:** 10.3352/jeehp.2023.20.6

**Published:** 2023-02-27

**Authors:** Ju Yoen Lee

**Affiliations:** Hanyang University School of Law, Seoul, Korea; Hallym University, Korea

**Keywords:** Authorship, Artificial intelligence, Chatbot, Copyright, Research ethics

## Abstract

At the end of 2022, the appearance of ChatGPT, an artificial intelligence (AI) chatbot with amazing writing ability, caused a great sensation in academia. The chatbot turned out to be very capable, but also capable of deception, and the news broke that several researchers had listed the chatbot (including its earlier version) as co-authors of their academic papers. In response, Nature and Science expressed their position that this chatbot cannot be listed as an author in the papers they publish. Since an AI chatbot is not a human being, in the current legal system, the text automatically generated by an AI chatbot cannot be a copyrighted work; thus, an AI chatbot cannot be an author of a copyrighted work. Current AI chatbots such as ChatGPT are much more advanced than search engines in that they produce original text, but they still remain at the level of a search engine in that they cannot take responsibility for their writing. For this reason, they also cannot be authors from the perspective of research ethics.

## Graphical abstract

[Fig f1-jeehp-20-06]

## Introduction

An artificial intelligence (AI) chatbot, called ChatGPT [1], which can generate human-like text, was released by Open AI in November 2022 and has since become a global issue. In education, concerns have arisen about students using this amazing chatbot to complete assignments [2]. It was also reported that this chatbot was listed in academic papers as a co-author [3]. Opinions were formulated about the need for guidelines for the use of AI chatbots in scientific writing [4]. In response to these concerns, *Nature* has added the following to its existing editorial policies [5,6]:

“Large Language Models (LLMs), such as ChatGPT, do not currently satisfy our authorship criteria. Notably an attribution of authorship carries with its accountability for the work, which cannot be effectively applied to LLMs. Use of an LLM should be properly documented in the Methods section (and if a Methods section is not available, in a suitable alternative part) of the manuscript.”

*Science* has also stated that it will specify in its license and editorial policy that ChatGPT-generated output cannot be used and attributed in papers [7].

A broader issue remains, however—can chatbots be authors of academic papers and, if not, why not? Even if, as *Nature* states, chatbots cannot be authors of academic papers now, what about more advanced chatbots in the future? Journal editors may wonder about this. Therefore, this article deals with the issue of AI chatbots as authors from the perspectives of law and research ethics.

### Ethics statement

As a literature-based legal study, approval by the Institutional Review Board and informed consent were not required.

### Study design

This study addresses the issue of AI chatbot authorship both from the legal and research ethics perspectives. It relied mainly on current law, judicial precedents, and other legal literature, which were searched in various legal databases.

## AI chatbot authorship from the perspective of copyright law

In November 1981, a computer program called Racter was named as the author of a prose text that was published in the magazine OMNI [8]. Subsequently, Racter’s book, the first written by a computer program, was published in 1984 [9]. Racter prompted substantial thought about AI and copyright issues [10]. Since then, with the development of the AI industry, many academic discussions have taken place about AI and authorship (and inventorship). The question is, can the authorship of AI be acknowledged from the perspective of current copyright law? Copyright offices and courts in many countries have generally expressed negative opinions on this issue. In some countries, the answer to this question can be found directly in their copyright statutes. For example, the Korean Copyright Act defines “a work” as “a creation that expresses the thoughts or feelings of a human being” and an “author” as “a person who creates a work? (Article 2-i, 2-ii) [11]. Therefore, according to the Act, anything created by a nonhuman being cannot be a copyrighted work, and a nonhuman being cannot be an author. In other words, it is self-evident that an AI chatbot cannot be an author under Korean law. In other countries, where the copyright statute does not directly address this issue, courts and copyright offices interpret their copyright statutes as endorsing the so-called “human authorship principle” (“human creator principle” may be a more accurate expression), which means that for a work to be copyrightable, it must be created by a human [12-17].

As a representative example, in the 2018 case of Feilin Law Firm v Baidu, the Beijing Internet Court of China articulated that the report automatically generated by the Wolters Kluwer Database in the inquest process is not a copyrighted work because it was not created by a natural person and the Wolters Kluwer Database cannot be recognized as its author [15]. Chinese copyright law does not explicitly state that the creator of a work must be a human being. Nonetheless, the court, on the grounds that AI does not have the capacity to have a right, held that originality alone is not sufficient for a work to be protected and a copyrighted work must be created by a natural person [15].

Courts in the United States have also protected only works created by natural persons. For example, in the Monkey Selfies case, an animal rights group argued on behalf of Naruto (a 6-year-old crested macaque) that the monkey was the author and copyright holder of the photos at issue. However, the US Court of Appeals dismissed the complaint on the grounds that monkeys are not humans and therefore lack statutory standing under the Copyright Act [16]. In 2022, the US Copyright Office endorsed the principle of human authorship by affirming its previous decision to reject copyright registration for a 2-dimensional artwork named “Entrance to Paradise,” which was allegedly automatically generated by an AI program named Creativity Machine [12].

In 2021, the Copyright Office of India and the Copyright Office of Canada both accepted a copyright registration application where an AI painting app named Raghav was listed as a co-author of a painting titled “Suryast” [18-20]. However, it is too early to determine that the copyright ability has been recognized for a work automatically generated by AI, or that the co-authorship of AI has been recognized. At first, the Indian Copyright Office rejected the application for copyright registration with Raghav as the sole author, but the application was accepted later, when Ankit Sahni, the owner of Raghav, applied for copyright registration with himself and Raghav as co-authors [18]. Above all, copyright is a nonregistered right, which means that copyright automatically arises at the same time as the creation of a work, regardless of any formalities, such as copyright notice or copyright registration (Article 5-2 of the Berne Convention for the Protection of Literary and Artistic Works, hereafter the “Berne Convention”) [21]. In other words, registration does not grant copyright; even if a work is registered with the Copyright Office, in the event of a legal dispute, the copyright of the work—and the status of the author and copyright owner—may be denied as a result of a court’s deliberation.

In the current copyright regime, the author of a work becomes the first copyright holder (Article 5-1 of the Berne Convention) [21]. In this regard, the fact that AI is denied a legal personality and cannot be a copyright holder serves as a strong argument that AI cannot be an author. Another argument is that AI cannot exercise rights by itself, even if certain rights are granted to AI. For example, AI cannot decide by itself whether to exercise moral rights, such as the right to make the work public, the right to claim authorship of the work, and the right to integrity of the work, which are inalienable and exclusive to the author, unlike the economic rights of a work. In this respect, it is clear that AI cannot be an author under the current copyright regime. In addition, in a similar vein, AI cannot be an inventor [22,23].

## Cases to be distinguished

Journal articles and books have, in some cases, been authored by an institution or a group, and in other cases, writing is published under a pseudonym. What distinguishes these situations from occasions when chatbots are listed as authors?

### Cases where an institution or organization is listed as an author

In some cases, the name of an institution or group is listed as the author of a book or academic paper. One may wonder whether this contradicts the principle of human authorship outlined above. In fact, it does not. Here, the institution or group named as the author may refer to all the natural per sons belonging to it, or it may refer to a work made for hire (Articles 2-xxxi and 9 of the Korean Copyright Act, Article 11-3 of the Chinese Copyright Act, Section 101 of the US Copyright Act [definition of a “work made for hire”], etc.) [11,24,25].

If all the people belonging to an institution or group contributed to the writing of a book or article, the name of the institution or group may be listed instead of listing all the names of the individuals. In this case, all the people belonging to the institution or group are considered co-authors. If a person employed by a research institution or research group writes an article as part of the business (i.e., research) of that institution or group, the institution or group may be the author or the first copyright holder as it is a work made for hire. A work made for hire means a work prepared by an employee within the scope of his or her employment. It should be noted that the concept and scope of a work made for hire may differ from country to country. In any case, it is common and preferable to list the names of all the individuals involved in writing the book or article in an appropriate place (such as in the acknowledgments, the author’s information section, or the copyright page).

What should not be overlooked here is that the persons who wrote the book or article mentioned above are humans (natural persons). In Feilin Law Firm v Baidu, the court held that the report at issue was a work made for hire of the plaintiff Feilin Law Firm because it was found that the human employees of the plaintiff created the report at issue with the “assistance” of the Wolters Kluwer Database. If, instead, the above-mentioned report had been automatically generated by the Wolters Kluwer Database, as the defendant Baidu argued, the copyright ability of the report would have been denied, and therefore the court would not have been able to recognize the report as a work made for hire of the plaintiff [15]. In Shenzhen Tencent v Shanghai Yingxun, another case in China, the court acknowledged by the same logic that an article on the stock exchange was a work made for hire of Tencent [17]. These cases show that the principle of human authorship or a human creator must be complied with even in the case of a work made for hire.

### Cases of publication under a pseudonym

A person may publish his or her writing under a pseudonym (for example, the name of a beloved pet). In fact, in the literary world, it is not unusual for authors to use pen names for various reasons [26]. In this case, the real author is known to the publisher but not to the public [26], and there is no intention to deceive the publisher or the public. From the perspective of copyright law, using a pen name or to maintain the anonymity of a work is also an exercise of the author’s right to claim authorship [27]. In the case of academic papers, it is usual and desirable to accurately provide the names and affiliations of the authors to ensure the reliability of the paper and promote academic discussion. However, in exceptional circumstances where it is necessary to publish an academic paper under a pseudonym or anonymity, it is not impossible to do so with the permission of the publisher.

It is necessary to distinguish between publishing one’s writing using a pseudonym and publishing an article under a fake author’s name to make a non-author appear to be the author. The latter is based on the intention to deceive the journal that decides to publish the article, as well as the entire academic community. It is a clear violation of research and publication ethics [28], and also a crime according to Article 137-1-i of the Korean Copyright Act [11].

## AI chatbot authorship from the perspective of research ethics

Aside from the discussion on copyright law, from the perspective of research and publication ethics, the question remains of whether an AI chatbot can become an author of an academic paper. The answer to this question is, “it all depends.”

The fact that AI cannot be an author under copyright law does not mean that an AI should never be listed as an author of an academic paper. This is because if a writing is not the work of a human, it may not be appropriate to attribute it to a human as an author.

Earlier, we saw the case of Racter, where an AI was actually attributed as the author. In the scientific community, a book authored by AI was published in 2019. The author of Lithium-Ion Batteries, introduced as the first machine-generated research book, is Beta Writer, an algorithm developed through a collaboration between Springer Nature and researchers at Goethe University [29].

From a legal point of view, writings generated by Racter and Beta Writer are not copyrighted works, and Racter and Beta Writer cannot be considered authors. Still, it was appropriate to publish the works under the names of “Racter” and “Beta Writer” because it would be against publishing ethics to publish such writings under the name of human beings. As the Beijing Internet Court mentioned as dicta in the case of Feilin Law Firm v Baidu, AI-generated outputs must not have a human being indicated as the author, whether the human being is the developer (owner) of the AI program or its user (a person who has rights and interests in the AI creation as determined by the court), and it must be indicated that the outputs were automatically generated by AI [15].

The publication of academic writing depends not on whether it is copyrighted, but on whether it can contribute to academia. As mentioned in the introduction to Lithium-Ion Batteries, written by one of the project directors, the reason why this book was published (i.e., the value of this book) did not lie in its content (i.e., the research results). In fact, the book contained many manifest flaws, such as grammatical errors. Rather, the real value of the book lay in the fact that “Beta Writer,” which is not a human being, wrote a book on scientific research, which was expected to promote related discussions and future research. Likewise, if an editor thinks that an academic paper that was generated by an AI chatbot has some academic value, he or she may allow the publication of the paper credited to ChatGPT.

Then, why did major journals such as Nature and Science declare that AI chatbots cannot be authors of articles published in their journals? The reason can be found in Nature’s editorial policies on authorship, which state, “[AI chatbots] do not currently satisfy our authorship criteria” (emphasis added) [5]. In other words, the reason why an AI chatbot cannot be an author is not just because AI chatbots are not human, but because the currently available AI chatbots do not meet the required qualifications for accountability. This also implies that an advanced AI chatbot in the future might meet the criteria for authorship of academic papers. It has also been pointed out that the fact that AI chatbots do not have the capacity to consent to the distribution of the paper is another reason why they cannot be considered authors [3], but this is only an argument from the perspective of copyright. From the perspective of research ethics, if an AI chatbot makes a significant contribution to research and can explain and prove the research results, it would be reasonable to recognize its authorship.

Today’s most advanced AI chatbot seems to be able to play the role of a research assistant in much the same way as a search engine. Whereas a search engine provides only search results (a list of related literature), an AI chatbot can be considered a more advanced research assistant in that it provides its own answers to users’ questions based on the related literature that it has learned. It is not reasonable to prevent a researcher from using a chatbot as a research tool and benefiting from the help it can provide, which would be similar to asking a researcher to perform arithmetic without a calculator. What is interesting is that ChatGPT, which has recently become a hot topic, cannot provide sources for its writings, and ChatGPT even has an unfortunate ability to provide fake information in a convincing way [30]. Therefore, AI chatbots such as the current ChatGPT are not “ideal” research assistants. A decent researcher would never fail to verify a text written by a research assistant who was good at writing, but also good at lying.

## Conclusion

The current AI chatbot cannot be the author of an academic paper, not only from the perspective of copyright law but also from the perspective of research ethics. Although researchers can use AI chatbots as research tools, they must be aware that AI chatbots can be competent but dangerous research assistants, and the authenticity of any AI-generated text must be verified. Researchers should always remember that although using AI chatbots is exciting and full of potential, it also comes with heavy responsibilities.

## Figures and Tables

**Figure f1-jeehp-20-06:**
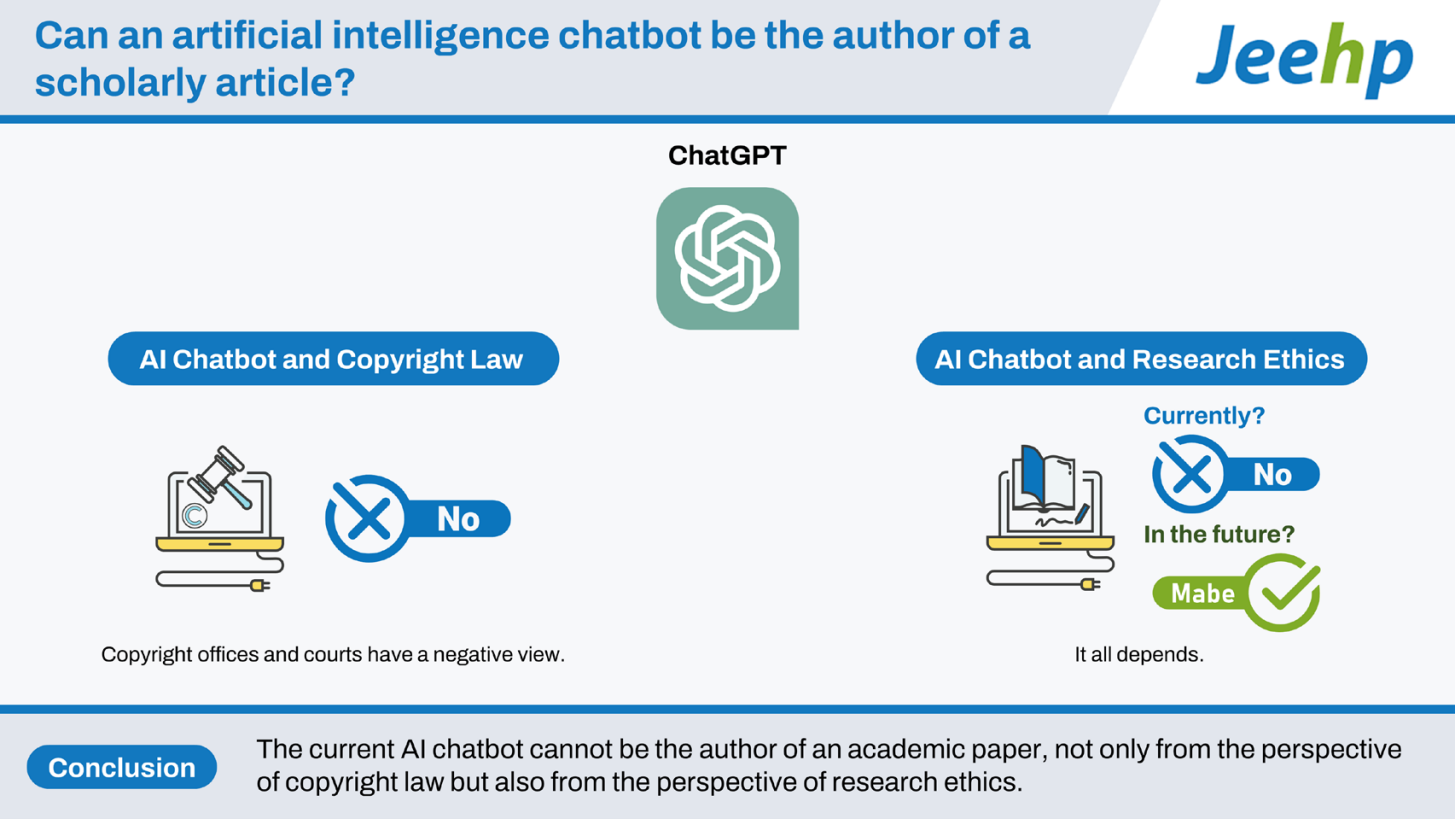


## Supplementary materials

Supplement 1. Audio recording of the abstract.
